# Midlife work ability and mobility limitation in old age among non-disability and disability retirees - a prospective study

**DOI:** 10.1186/s12889-016-2846-y

**Published:** 2016-02-16

**Authors:** Monika E. von Bonsdorff, Taina Rantanen, Timo Törmäkangas, Jenni Kulmala, Timo Hinrichs, Jorma Seitsamo, Clas-Håkan Nygård, Juhani Ilmarinen, Mikaela B. von Bonsdorff

**Affiliations:** Gerontology Research Center and Department of Health Sciences, University of Jyväskylä, PO Box 35, Jyväskylä, Finland; Folkhälsan Research Center, Helsinki, Finland; Chronic Disease Prevention Unit, National Institute for Health and Welfare, Helsinki, Finland; School of Health Care and Social Work, Seinäjoki University of Applied Sciences, Seinäjoki, Finland; Division of Sports and Exercise Medicine, Department of Sport, Exercise and Health, University of Basel, Basel, Switzerland; Finnish Institute of Occupational Health, Helsinki, Finland; Gerontology Research Center and School of Health Sciences, University of Tampere, Tampere, Finland; University of Eastern Finland, Kuopio, Finland

**Keywords:** Work ability, Disability retirement, Mobility limitation, Aging, Municipal employees

## Abstract

**Background:**

Little is known about the wellbeing and mobility limitation of older disability retirees. Personal and environmental factors, such as time spent in working life, may either exacerbate or mitigate the onset of mobility limitation in general population.

We aimed to study perceived midlife work ability as a determinant of self-reported mobility limitation in old age among municipal employees who transitioned into non-disability and disability retirement.

**Methods:**

4329 participants of the Finnish Longitudinal Study of Municipal Employees (FLAME) had retired during January 1985 and July 2000. They had data on retirement, perceived work ability in 1985, and self-reported mobility limitation (non-disability retirement *n* = 2870, men 39 %; and diagnose-specific disability retirement *n* = 1459, men 48 %). Self-reported mobility was measured in 1985, 1992, 1997 and 2009. The latest score available was used to assess the number of mobility limitation. Work ability was measured by asking the respondents to evaluate their current work ability against their lifetime best in 1985. Incidence rate ratios (IRRs) and 95 % confidence intervals (CIs) for work ability predicting mobility limitation in non-disability and diagnose-specific disability retirement groups were calculated using Poisson regression models.

**Results:**

The prevalence of mobility limitation for those who transitioned into non-disability retirement (Incidence Rate, IR = 0.45, 95 % CI = 0.44–0.46) was lower compared to those who retired due to disability (IR = 0.65, CI = 0.63–0.66). A one-point increase in the work ability score decreased the risk for having one more mobility limitation among non-disability and all diagnose-specific retirement groups (musculoskeletal disease, cardiovascular disease, mental disorder, and other diseases).

**Conclusions:**

Better midlife work ability may protect from old age mobility limitation among those who retire due to non-disability and disability. Promoting work ability in midlife may lead to more independent, active aging, regardless of type of retirement.

## Background

Optimal mobility, which can be defined as relative ease and freedom of movement in all of its forms, is a central part of healthy aging [1]. It has positive consequences both for the aging individual and their communities in terms of maintaining the ability to carry out tasks related to daily activities [2] and decreased need of health care services [1, 3]. The longitudinal paths leading to and thorough mobility limitation can be understood by using the disablement process model [4]. According to this model, functional limitation, such as mobility limitation, is preceded by 1) impairment in the form of dysfunctions in specific body systems caused by pathology, such as diseases or injuries, and 2) risk factors related to e.g. lifestyle, the environment or psychological factors.

Multifactorial causes, which underlie mobility limitation, include comorbidity [5], as well as other individual, lifestyle-, environment, behavioral, and social risk factors [6–10]. Furthermore, the link between midlife occupational physical activity [11], as well as work stress [12], and old age mobility limitation has been studied. Still, little is known about the role of working life and work history, along with possible work life exposures, even if it is plausible that personal and environmental factors, such as time spent in working life, may either exacerbate or mitigate the course and the nature of the disablement process [4]. Work ability, which is a central concept for older employees, indicates the balance between employees’ resources and corresponding job demands [13, 14]. While this concept has to our knowledge not previously been linked with old age mobility limitation, individuals with poor work ability in midlife have higher risk of disability pension [15, 16], as well as old age disability and mortality [14].

The incidence of disability pension continues to be high in many European countries, despite improvements in health and increases in life expectancy in last decades [17]. Still, little is known about the life situation of individuals who were granted disability pension [18]. In 2013, 28 % of Finnish retirees transitioned into disability retirement, mainly due to mental illnesses, musculoskeletal disorders and cardiovascular disease (CVD) [19]. Together with a high number of chronic diseases, excessive work strain not only predicts early exit from working life via disability retirement [15, 16], but may also be a risk factor for the onset of mobility limitation [4, 10] and subsequent loss of mobility in older age [5]. Preventing disability retirement, as well as maintaining the ability to function among individuals who are at high risk for mobility decline by identifying work-related factors that may decrease the prevalence of mobility limitation in older age, are of importance for the public health.

Our aim was to investigate longitudinally midlife work ability as a determinant of mobility limitation among municipal employees, who transitioned into non-disability or diagnose-specific disability retirement during the 28-year follow-up. Based on previous findings on mobility limitation [5] and old age health outcomes among disability retirees [18, 20, 21], we expected primarily to find a higher incidence of old age mobility limitation among those who retired due to musculoskeletal disease or CVD, compared to non-disability retirees. As work ability is built on individuals’ physical, psychological, and social resources [13], we expected better work ability to decrease the risk of subsequent mobility limitation among non-disability and even among disability retirees [11, 12].

## Methods

### Participants

Data come from the Finnish Longitudinal Study on Municipal Employees (FLAME), conducted by the Finnish Institute of Occupational Health. At baseline in 1981, 6257 municipal sector employees aged 44–58-years working in various municipal occupations were randomly chosen from municipalities across Finland. They were followed up in 1985, 1992, 1997, and 2009 [14, 22]. The study, which focuses on work, health, and lifestyle factors, is on-going with register-based data on mortality available until end of July, 2009.

For the current study, we included all participants with data on work ability in 1985, type and date of formal retirement, along with self-reported information on post-retirement mobility limitation. This left us with a sample of 4329 participants (42.1 % men). Of the 1928 participants excluded from this study, 705 had no post-retirement data available on mobility limitation, 18 died before retirement, 648 had missing data on retirement or disability retirement diagnose (due to e.g. migration before retirement), 410 on mobility limitation, and 147 on both retirement and mobility limitation. Compared to the effective sample in the current study, those with missing register data, were older, reported poorer work ability, were more often men, blue-collar (versus upper or lower white-collar) employees, not married, and reported more smoking and drinking, less physical activity, and more CVD, musculoskeletal, metabolic, and mental disease. This study was conducted among adult individuals with the approval of the Finnish Institute of Occupational Health Ethical Committee and conforms to the principles of the Declaration of Helsinki.

## Measures

### Work ability

Work ability was assessed in 1985, as a subjective evaluation of present work ability compared to lifetime best, with the question: ‘Assume that your work ability at its best has a value of 10 points. What score would you give your current work ability?’ This question is part of the seven-item Work Ability Index (WAI) [23] and has been found to capture most of the individual differences of the index [16]. WAI is accepted as a world-wide measure of work ability and it was developed at the Finnish Institute of Occupational Health in the 1980’s [13].

### Non-disability and diagnose-specific disability retirement

Finnish Centre for Pensions provided data on date and type of retirement and primary diagnosis for disability retirement, which was linked to the survey data using a unique personal identification number. All participants retired during January 1985 and July 2000. During the time of their retirement, statutory retirement age for municipal employees was 63 years, with the exception of special groups (e.g. pre-school teachers, bus drivers and nurses for which it was somewhat lower at 55 or 58 years) [22]. Transitioning into retirement was at that time a permanent decision, with no possibility to further participate in paid work. Besides old age retirement, employees could apply for an individual early retirement at the age of 58 years. This was usually done for reasons such as health problems, a long work career, lack of skills required in the job or working conditions, and lead to a permanent reduction in the pension level [24]. Together these two forms of retirement comprised the “non-disability retirement” group in the current study.

Disability retirement could be applied for before reaching the statutory retirement age, if the employee was due to a medically confirmed illness unable to continue working, even after periods of rehabilitation, re-education or assistance [25]. Following the International Classification of Diseases (ICD 8–10), we classified disability retirements into the following groups: diseases of the musculoskeletal system (ICD 8–9 codes 710–739 and ICD 10 codes M00-M99), diseases of the cardiovascular system (ICD 8–9 codes 390–459 and ICD 10 codesI00-I02), mental disorders (ICD 8–9 codes 290–319 and ICD 10 codes F00-F99) and all other diagnoses which included e.g. diseases of the respiratory or nervous systems and injuries or poisoning.

### Mobility limitation

Mobility limitation were assessed using the latest available measure from self-reported questionnaire data collected in 1985, 1992, 1997, and 2009, described in detail elsewhere [11, 12]. The assessment of mobility limitation included eight questions, thereof three questions on walking and moving (walking 2 km, running 100 m, and climbing three flights of stairs); two questions on changing and maintaining body position (squatting down and standing up again, and bending down deep e.g., to reach the feet); and three questions on carrying, moving, and handling objects (lifting and carrying heavy loads of more than 10 kg, performing precise movements with hands and fingers e.g., potato peeling or using a screwdriver, and lifting hands over the head). The level of difficulties respondents may encounter while performing these tasks were also evaluated. If the respondent indicated having “no difficulty”, the score for each individual item was coded as 0. The corresponding score was coded as 1 for those, who indicated having at least “some difficulties”. A summary score was then calculated, ranging from 0 (no difficulty in any activity) to 8 (at least some difficulties in all activities) [11, 12]. While this score does not take the severity of mobility limitation into account for the individual items, it has proven to be a reliable measure of mobility limitation [26–28].

### Mortality

Mortality dates were obtained from the Finnish National Population Register between January 1981 and June 2009. Age until death or censoring on 31st July, 2009 was used as measure of survival follow-up time.

### Covariates

Demographic information on respondents’ age, gender and marital status (married or cohabiting vs. other) was obtained at baseline. Based on objective assessments of job characteristics at baseline, respondents were classified into the following occupational classes: blue-collar (e.g. maintenance, home care), lower white-collar (e.g. transport work, dental care), and upper white-collar (administrator, physician, teacher) [22]. The presence of baseline self-reported physician diagnosed major chronic diseases included musculoskeletal diseases (e.g. arthritis, degenerative back diseases), cardiovascular diseases (e.g. arterial hypertension, angina pectoris), metabolic disorders (e.g. diabetes, obesity), mental disorders (e.g. depression, anxiety), and cancer (benign or malignant). Lifestyle factors assessed at baseline included smoking status (never smoked / ever smoked), alcohol consumption (at least once a week / less) and physical activity during previous year (vigorous activity at least once a week / less).

### Statistical analyses

Baseline descriptive statistics according to type of retirement are reported as percentages for categorical values and means and standard deviations for continuous variables.

Two models for each retirement group (non-disability, disability due to CVD, musculoskeletal diseases, mental disorders, and other diagnoses) on work ability as a predictor of old age mobility limitation were estimated. First, we modelled mobility in a Poisson regression model adjusting for age as an offset and gender. In the second model mobility was a Poisson-distributed outcome with adjustment made for age, gender, marital status, occupational class, chronic diseases, and lifestyle factors while mortality risk was adjusted by a random effect obtained from a simultaneous Cox regression model. In addition to the join models for both genders, we present separate models for men and women. Missing data in independent variables were assumed to be missing at random (MAR), which was accounted for in the likelihood construction for the estimation of parameters in Mplus version 7. Further details of the method can be found in Hinrichs et al. [11]. Results are presented as incident rate ratios (IRRs) with 95 % confidence intervals (CIs). Percentage decrease in IRR was calculated as: 100*(exp(−log(IRR))-1). This statistic expresses risk decrease in mobility limitations for a unit increase in the workability index.

Descriptive statistics according to type of retirement were analyzed using SPSS 18.0 (IBM Corporation, Somers, NY, USA). Mplus Version 7 was used to calculate the parameters of the Poisson models [29]. CIs of incidence rates were calculated using the epiR package Version 0.9.48 in the R programming environment Version 2.15.2 (R Foundation for Statistical Computing, Vienna, Austria), which is based on methodology described elsewhere [30].

## Results

Approximately two thirds of the participants (*n* = 2870) transitioned into non-disability retirement (39 % men), while one third (*n* = 1459, 48 % men) retired due to disability. Of these disability retirees, 714 (49 %) retired due to diseases of the musculoskeletal system, 268 (18 %) due to diseases of the circulatory system 208 (14 %) due to mental disorders, and 269 (19 %) due to all other diagnoses. Due to gender differences in type of retirement (*χ*^2^ = 98.750, df = 4, *p* < 0.001), the IRRs for old age mobility limitation by retirement groups were calculated separately for men and women. Sixty-nine percent of those who retired due to CVD and 35 % of those who retired due to mental disorders were men.

Baseline characteristics, mean of work ability measured in 1985, and the IR of old age mobility limitation according to type of retirement for all participants are presented in Table 1. Compared to non-disability retirees, those who retired due to disability were younger, more often men, married or cohabiting, blue-collar employees, suffered more frequently from major chronic illnesses, smoked and used alcohol more frequently, and were physically less active. Furthermore, those who retired due to disability reported poorer work ability in 1985 (mean = 5.8, sd = 2.7), compared to non-disability retirees (mean = 7.4, sd = 1.6, *t* = 19.981, df = 1836, *p =* .0.001). On average, the IR of mobility limitation for non-disability retirees was 0.45 (95 % CI = 0.44–0.46) and for disability retirees 0.65 (95 % CI = 0.63–0.66). On average, the last mobility limitation was measured at age 74.1 (sd = 6.8) for non-disability retirees and at age 71.2 (sd = 7.4) for disability retirees (*t =* 12.677, df = 2710, *p.* < 0.001). The time of mobility limitation measurement (in 1985, 1992, 1997, or 2009) by type of retirement is presented in Table 2.

**Table 1:** Baseline characteristics, mobility limitation, and work ability in year 1985 according to type of retirement for all participants (percentages unless stated otherwise)

	All participants *n* = 4329	Non-disability retirement *n* = 2870	Disability retirement, All causes *n* = 1459	Disability retirement, Musculoskel. *n* = 714	Disability retirement, Cardiovascular *n* = 268	Disability retirement, Mental disor. *n* = 208	Disability retirement Others *n* = 269
Baseline age (mean, SD)	50.1 (3.4)	51.1 (3.5)	49.5 (3.0)	49.5 (3.1)	49.5 (2.9)	48.9 (2.9)	49.5 (3.1)
Married/cohabiting (vs. single)	76	74	80	81	87	68	80
Men	42	39	48	43	68	35	53
Occupational class							
Upper white-collars	24	25	20	12	20	40	27
Lower white-collars	36	39	28	31	24	37	25
Blue-collars	40	35	51	57	56	23	48
Musculoskeletal disease	37	33	47	56	38	35	40
Cardiovascular disease	20	17	25	22	42	21	19
Metabolic disease	6	5	8	7	8	9	7
Mental disorder	2	1	3	2	0	8	3
Cancer	1	4	6	6	4	7	5
Never smoked	58	62	52	54	45	62	48
Alcohol consumption ≥ 1 per week	9	8	12	12	15	10	8
Vigorous physical activity ≥ 1 per week	50	53	44	43	46	42	47
Mobility limitation (Incidence Rate, 95 % CIs) ^a^	0.51 (0.51–0.52)	0.45 (0.44–0.46)	0.65 (0.63–0.66)	0.66 (0.64–0.68)	0.67 (0.64–0.71)	0.54 (0.51–0.58)	0.66 (0.63–0.70)
Age at last mobility limitation measurement, (mean, SD)	73.1 (7.1)	74.1 (6.8)	71.2 (7.4)	71.5 (7.4)	70.4 (7.7)	71.8 (7.3)	70.5 (7.2)
Work ability in 1985, (mean, SD)	6.8 (2.2)	7.4 (1.6)	5.8 (2.7)	5.7 (2.6)	5.5 (2.9)	5.9 (2.7)	5.9 (2.7)
Mortality during follow-up (Incidence Rate, 95 % CIs)^b^	3.26 (3.07–3.46)	4.04 (3.68–4.43)	2.87 (2.66–3.10)	3.42 (2.97–3.95)	5.52 (4.58–6.65)	3.31 (2.53–4.34)	4.81 (3.95–5.87)

*SD* standard deviation, ^a^Incidence Rate (IR), number of limitation per 10 person-years, ^b^Number of deaths per 1000 person-year

**Table 2:** Last mobility limitation measurement by type of retirement (percentages)

Year of mobility limitation measurement	All participants *n* = 4329	Non-disability retirement *n* = 2870	Disability retirement, All causes *n* = 1459	Disability retirement, Musculoskel.*n* = 714	Disability retirement, Cardiovascular *n* = 268	Disability retirement, Mental disor. *n* = 208	Disability retirement Others *n* = 269
Year 1985	2.5	2.0	3.3	1.8	6.7	2.4	4.5
Year 1992	10.7	9.4	13.4	13.3	14.9	9.1	15.9
Year 1997	29.3	28.5	30.8	31.0	32.1	28.4	31.2
Year 2009	57.5	52.1	52.5	53.9	46.3	60.1	48.7

Of the effective sample, those who died during the follow-up (*n* = 1087) were older at baseline, had poorer work ability and were more likely to be men and blue-collar employees. Furthermore, they suffered more frequently from CVD, musculoskeletal and metabolic diseases, smoked more, used alcohol more frequently, and were physically less active.

IRRs and their 95 % CIs for work ability predicting old age mobility limitation are presented in Table 3. A one-point increase in the work ability score decreased the risk for having one more mobility limitation in all retirement groups, with age-adjusted risk decrease ranging between 13.6 and 4.2 %. The highest decrease was found for those who transitioned into non-disability retirement (IRR = 0.88, 95 % CI = 0.87–0.89). Further adjustment for chronic illnesses, lifestyle factors, and mortality risk attenuated little these risks among men and women (percentages ranging between 9.9 and 3.1).

**Table 3:** Incidence rate ratios and their 95 % confidence intervals for work ability as a predictor of mobility limitation. Unadjusted and mortality- and covariate-adjusted models presented for all, men and women

	All (*n* = 4329)			Men (*n* = 1826)			Women (*n* = 2503)		
		95 % Confidence interval			95 % Confidence interval			95 % Confidence interval	
	IRR	Lower	Upper	IRR	Lower	Upper	IRR	Lower	Upper
Unadjusted Model^a^									
Non-disability	**0.88**	**0.87**	**0.89**	**0.87**	**0.85**	**0.88**	**0.89**	**0.88**	**0.91**
CVD	**0.94**	**0.92**	**0.96**	**0.93**	**0.91**	**0.95**	0.97	0.93	1.01
Musculoskeletal	**0.95**	**0.94**	**0.96**	**0.95**	**0.93**	**0.97**	**0.95**	**0.94**	**0.97**
Mental disorder	**0.93**	**0.90**	**0.95**	**0.90**	**0.86**	**0.94**	**0.94**	**0.91**	**0.97**
Other	**0.96**	**0.94**	**0.97**	**0.97**	**0.94**	**0.99**	**0.94**	**0.91**	**0.97**
Mortality and covariate adjusted Model^b^ ^c^									
Non-disability	**0.91**	**0.90**	**0.92**	**0.88**	**0.85**	**0.91**	**0.92**	**0.91**	**0.93**
CVD	**0.95**	**0.93**	**0.98**	**0.95**	**0.92**	**0.98**	0.97	0.93	1.02
Musculoskeletal	**0.96**	**0.95**	**0.97**	**0.96**	**0.94**	**0.99**	**0.96**	**0.94**	**0.98**
Mental disorders	**0.96**	**0.92**	**0.99**	0.94	0.88	1.01	0.97	0.93	1.01
Other	**0.97**	**0.95**	**0.99**	0.98	0.95	1.02	**0.95**	**0.92**	**0.99**

Bold typeface indicates effect significant at the level of *p* ≤ 0.05

^a^Offset term for time in follow-up was used for the mobility outcome

^b^ Covariates included in the model were marital status, occupational class, chronic illnesses, and lifestyle factors

^c^Missing data in covariates adjusted for by maximum likelihood estimation under the assumption of missing-at-random

Due to a statistically significant interaction between gender and retirement type on mobility limitation, further analyses were stratified by gender. In the non-disability retirement and disability retirement due to musculoskeletal disease groups, a one-point increase in the work ability score decreased the risk of having one more mobility limitation (13.6 % and 4.2 % for men, 8.7 % and 4.2 % for women, respectively). Further, for men, a similar risk-decrease was found in the disability due to CVD-retirement group (IRR = 0.95, CI 95 % = 0.92–0.98), as well as for women in the disability due to other diagnoses-retirement group (IRR = 0.95, CI 95 % = 0.92–0.99).

## Discussion

This prospective large-scale study on municipal employees showed that the incidence rate of mobility limitation was higher among those who transitioned into disability retirement due to CVD, musculoskeletal and other diseases, compared to non-disability retirees. Furthermore, better work ability in midlife was associated with a decreased risk of mobility limitation among non-disability and disability retirees in all diagnose-specific groups. Allowing for main chronic illnesses, lifestyle factors and mortality risk did not markedly attenuate these associations. These findings are significant, as mobility limitation often leads to old age disability [31].

The link between better midlife work ability and lower incidence rate of old age mobility limitation among non-disability and disability retirees supports previous studies which have found vigorous occupational physical activity [11] and higher work stress [12] in midlife to be associated with old age mobility limitation. The current findings may be the result of several factors, characteristic to the concept of work ability [13] and the disablement process [4]. In addition to health and lifestyle, several factors linking midlife work ability to old age mobility limitation can be identified both among non-disability and disability retirees.

*First*, those individuals who report good work ability are likely to possess high levels of physical, psychological, and social resources in midlife [13, 15]. While these resources help employees to succeed in their jobs, they may also buffer the onset of disability [26], even among those who retire due to disability. Work-related risk factors for disability retirement are e.g. physically demanding work and low job control [32, 33]. In the face of chronic illness and subsequent disability retirement, better midlife work ability may indicate more resources, including psychological and social, for the aging individual to fall back on. These resources may postpone the onset of mobility limitation, even among those who have retired due to e.g. musculoskeletal or cardiovascular disease. Furthermore, according to the model of the disablement process [4], the density of the social network may be an important extra-individual determinant of the onset of mobility limitation. Ways to support work ability include decreasing the physical work load, making adjustments to work-rest schedules, and introducing age-management practices, flexible working time schedules, and teamwork [34].

*Second*, the balance between employee resources and job demands, i.e. the absence of prolonged occupational physical and mental strain in midlife, may protect employees from old age mobility limitation [11, 12]. In fact, the positive effect of occupational activity and work-engagement later in life may be reflected in old age health outcomes, such as disability or mobility [35, 36]. Especially among non-disability retirees, maintaining activity through participating in working life may help ensure mobility in old age [37]. However, it must be acknowledged that work ability is a concept which indicates the balance between employee resources and respective work demands [13]. Hence, an individual with good resources in an extremely high-strain job may report poor work ability. Vice versa, an individual with low resources in an extremely light job may report good work ability.

*Finally*, as work ability is partly built on employees’ attitudes, motivation and values [13], it is plausible that better work ability, stemming from higher motivation for participation and positive attitudes towards work, is reflected in old age mobility. More specifically, these work-related attitudes, motivations and values may be reflected in intra-individual factors significant in terms of slowing down the disablement process [4], such as positive attitude, emotional vigor, locus of control, and cognitive adaptation to one’s situation. Thus, even individuals who retire due to disability, may in terms of maintaining mobility in old age, benefit from positive midlife work attitudes and a sense of competence.

The current findings indicate some gender-differences according to type of retirement and the association between work ability and mobility limitation. Compared to women, men were more likely to retire due to CVD and less likely to retire due to other diseases, such as neurological or respiratory diseases. According to Finnish Center for Pensions statistics men were more likely to retire due to disability caused by CVD compared to women (9.1 % vs. 4.5 %, out of all those retired due to disability in 2013) [19]. Furthermore, better work ability decreased the risk for old age mobility limitation among men who retired due to CVD and among women, who retired due to other diseases, such as neurological and respiratory diseases. These findings may indicate that for men, old age mobility limitation may be linked with work-related factors, particularly poor work ability in middle age. The association between work strain in midlife, similar to poor work ability, and negative health outcomes in old age is stronger for men compared to women [38].

Finally, while better work ability decreased the risk for old age mobility limitation among those who retired due to mental disorders in the combined model for men and women, this association was not found in the models stratified by gender. This finding may be caused by the low number of participants in this disability retirement group (*n* = 208, men *n* = 72). The similar type of association between work ability and mobility limitation for all participants and in the gender-stratified models, along with the wider CIs, indicate that these findings may be power-related. Besides the low prevalence of mobility limitation in this disability retirement group and the low number of individuals, there may be other factors explaining this finding. Notably, the majority of those who retired due to mental diseases worked in higher white-collar jobs. This may not only explain the relatively low mobility limitation incidence (IR = 0.54, CI = 0.51–0.58), compared to other disability retirement groups, but also affect the role of work ability on old age mobility limitation.

This study was based on a large-scale population-based dataset consisting of a wide variety of municipal occupations, with both genders represented. The long follow-up time allowed us to explore midlife work ability in association with old age mobility limitation. Common to all follow-up studies, there are some limitations that need to be addressed. First, while self-reported mobility limitation and work ability were measured with widely accepted and validated scales [26–28, 39], some self-report bias might have influenced the results. Retirement and mortality data were retrieved from national registers. Second, as mobility limitation was not available at baseline, we cannot rule out the possibility that some participants had some mobility limitation at baseline. However, because all participants were occupationally active at baseline and were likely to have only a minimal level on mobility limitation at that time, we do not believe that this influenced the results markedly. Also, controlling for age at last mobility limitation measurement changed little the regression results. Third, common to corresponding long-term prospective designs, this study may be influenced by selective dropout. The ‘healthy worker survivor effect’ is an ongoing process where those who stay in a specific occupation tend to be healthier than those who exit employment [40]. As a result of this potential selection in the data, our results may be an underestimation of the incidence of mobility limitation in old age. Finally, participants of the current study were occupationally active municipal sector employees, which should be considered when generalizing the present results on a population level.

## Conclusions

Taken together, the current findings indicate that the balance between employee’s resources and work demands in midlife has independent, long-term effects on old age mobility, as better midlife work ability may protect from old age mobility limitation among those who retire due to non-disability and disability. Promoting work ability in midlife may lead to more independent, active aging, regardless of type of retirement.

## Abbreviations

CI: 95 % confidence interval
CVD: cardiovascular disease
FLAME: Finnish longitudinal study of municipal employees
ICD: international classification of diseases
IRR: incidence rate ratio
WAI: work ability index
